# The Brooklyn Dental Society

**Published:** 1871-10

**Authors:** 


### Editorial.

### THE BROOKLYN DENTAL SOCIETY.

It was a very grateful privilege we enjoyed recently, in being present at the Brooklyn Dental Society, at its annual meeting, to see between thirty and forty members of the profession in one city in council, met for the consideration and discussion of matters pertaining to the best interests of the profession. That this is done every month, argues well not only for that locality, but for the profession of the whole country. From that number of educated, earnest, working men, meeting regularly and laboring, diligently for the promotion of science, an influence will go out that will be wide reaching and operative, in breaking down prejudice and selfishness, and opening the way for the entrance of light and knowledge. Harmony and unanimity of feeling characterize the profession in Brooklyn, an illustration of which is shown in the establishment and maintenance of a dental hospital by the Brooklyn Dental Society.

Among the interesting matters that were presented, was a decided improvement in working the "Celluloid Base," for artificial dentures, by Dr. Beatrice, of New York. His method simplifies the work very much, and is very efficient. Long may the Brooklyn Dental Society live, and greatly may it prosper. T.

